# Endovascular Abdominal Aneurysm Repair in Women: What are the Differences Between the Genders?

**DOI:** 10.5935/1678-9741.20160047

**Published:** 2016

**Authors:** Rui Machado, Gabriela Teixeira, Pedro Oliveira, Luís Loureiro, Carlos Pereira, Rui Almeida

**Affiliations:** 1 Hospital de Santo António - Centro Hospitalar do Porto, Porto, Portugal; 2 Instituto de Ciências Biomédicas Abel Salazar (ICBAS), Porto, Portugal

**Keywords:** Aortic Aneurysm, Abdominal, Endovascular Procedures, Women

## Abstract

**INTRODUCTION:**

Abdominal aortic aneurysm has a lower incidence in the female population, but a higher complication rate. It was been hypothesized that some anatomical differences of abdominal aortic aneurysm in women could be responsible for that. We proposed to analyze our data to understand the differences in the clinical and anatomical characteristics and the outcomes of patients undergoing endovascular aneurysm repair, according to gender.

**METHODS:**

A retrospective analysis of patients undergoing endovascular aneurysm repair between 2001-2013 was performed. Patients were divided according gender and evaluated regarding age, atherosclerotic risk factors, aneurysm anatomic features, endograft type, anesthesic risk classification, length of stay, reinterventions and mortality. Two statistical studies were performed, first comparing women and men (Group A) and a second one comparing women and men, adjusted by age (Group B).

**RESULTS:**

Of the 171 patients, only 5.8% (n=10) were females. Women were older (*P*<0.05) and the number of women with no atherosclerotic risk factor was significantly higher. The comparison adjusted by age revealed women with statistically less smoking history, less cerebrovascular disease and ischemic heart disease. Women had a trend to more complex anatomy, with more iliac intern artery aneurysms, larger aneurysm diameter and neck angulations statistically more elevated. No other variables were statistically different between age groups, neither reintervention nor mortality rates.

**CONCLUSION:**

Our study showed a clear difference in the clinical characteristics of women. The female population was statistically older, and when compared with men adjusted by age, had less atherosclerotic risk factors and less target organ disease. Women showed a more complex anatomy but with the same outcomes.

**Table:** 

**Abbreviations, acronyms & symbols**	
AAA	= Abdominal aortic aneurysm
ASA	= American Society of Anesthesiologists
BMI	= Body mass index
EVAR	= Endovascular aneurysm repair

## INTRODUCTION

Abdominal aortic aneurysm (AAA) has a lower incidence in the female population^[1]^, but a higher complication rate^[2-5]^, especially a higher risk of rupture^[6]^. It was been hypothesized that some different clinical and anatomical characteristics of AAAs in women could be responsible for this higher rate of complications and worse results after treatment.

As the incidence of AAAs is higher in men, the therapeutic indications for AAAs in women are generally extrapolated from studies that have a small number of women, and so they are based mostly on results obtained from men.

We decided to evaluate in our center if gender had influence on the clinical, anatomical characteristics and outcomes after endovascular aneurysm repair (EVAR).

## METHODS

A retrospective analysis of our database of patients undergoing EVAR was performed. A total of 171 patients treated between October 2001 and December 2013 and with a diagnosis of infrarenal aortic or aortoiliac aneurysms were included. The adopted surgical criteria were degenerative fusiform aneurysm diameter >5 cm, aortic aneurysm <5 cm associated with common iliac artery aneurysm >3 cm, saccular aneurysms, dissecting aneurysms and pseudoaneurysms. Patients with a diagnosis of ruptured abdominal aneurysm were excluded.

Patients were divided according gender and evaluated regarding age, atherosclerotic risk factors (hypertension, diabetes, smoke history, dyslipidemia), body mass index (BMI), comorbidities (ischemic heart disease, valvular heart disease, heart failure, cardiac arrhythmia, vascular neurological disease, chronic obstructive pulmonary disease, respiratory failure, chronic kidney disease), aneurysm morphology (aortic, right aortoiliac, left aortoiliac, bilateral aortoiliac), aneurysm diameter, neck form (conical, reverse conical, cylindrical, other), neck characteristics (diameter, calcification, thrombus, angulation), iliac morphology (tortuosity and diameter), anatomical risks, internal iliac artery aneurysms, endograft type, American Society of Anesthesiologists (ASA) classification, anesthetic technique, length of stay, reinterventions, and mortality.

Two statistical analysis were performed, the first comparing women (n=10) and men (n=161) (Group A), and the second comparing women (n=10) and an age-adjusted subgroup of men (n=47) (Group B). The statistical analysis included t-tests for two independent samples, analyses of variance in the case of several groups, and chi-square tests for the comparison of proportions concerning categorical variables. Nonparametric tests were used when the normality or homogeneity of variances was not observed. All the analyses were performed with IBM SPSS Statistics, version 22; the statistical significance for twosided tests was assumed to be *P*<0.05.

## RESULTS

Of the 171 patients, 94.2% (n=161) were males and 5.8% (n=10) were females. We analyzed patients undergoing open surgery during the same period and found that 5% were women, with no statistical difference between the proportions (*P*=0.478). The mean age of patients undergoing EVAR was 74.1 years, with the median of 75 years and a standard deviation of 8.9 (min.: 38, max.: 93). In the male population, the mean age was 73.8±8.9 years, while women's was 79.8±6.9 years. The female population was statistically older (*P*=0.037).

Clinical characteristics comparison between gender is shown in Table 1. In group A, women had statistically fewer association with smoking history, association of atherosclerotic risk factors, and fewer vascular neurological disease, and a trend to a fewer arterial hypertension. In Group B, women had statistically fewer association with smoking history, ischemic heart disease, vascular neurological disease, and a trend to fewer arterial hypertension and atherosclerotic risk factors association.

**Table 1:** Comparison of clinical characteristics between women and men (Groups A and B).

			**Group A (general)**		**SS**	**Group B (adjusted by age)**		**SS**
			**Men (n=161)**	**Women (n=10)**		**Men (n=47)**	**Women (n=10)**	
Body Mass Index		BMI < 25	41.8%	33.3%	N	52.3%	33.3%	N
		BMI 25-30	36.2%	66.7%		34.1%	66.7%	
		BMI> 30	22%	0		13.6%	0	
Hypertension			85.7%	40%	0.053	87.2%	40%	0.062
Diabetes			18.1%	20%	N	23.9%	20%	N
Dyslipidemia			68.1%	60%	N	73.9%	60%	N
Smokers		Active Smokers	17.5%	0	Y	13%	0	Y
		Former Smokers	61.9%	10%	Y	65.2%	10%	Y
		No Smokers	31.4%	90%	Y	21.8%	90%	Y
Peripheral Arterial Disease			18.1%	81.9%	N	23.9%	20%	N
Atherosclerotic risk factors association		No risk factor	8.1%	30%	Y	8.5%	30%	0.07
		1-2 risk factors	24.2%	40%		21.3%	40%	
		3-4 risk factors	56.5%	30%		63.8%	30%	
		≥ 5 risk factors	11.2%	0		6.4%	0	
Ischemic Heart Disease			54.50%	30%	N	68.9%	30%	Y
Valvular Heart Disease			26.70%	33.3%	N	31.6%	33.3%	N
Heart failure			43.90%	60%	N	54.5%	60%	N
Cardiac arrhythmia			36.50%	50%	N	52%	50%	N
Vascular neurological disease			12.70%	0	Y	14.9%	0	Y
COPD			24.30%	20%	N	26.7%	20%	N
Respiratory Failure			5.30%	10%	N	11.1%	10%	N
Chronic Kidney Disease			22.10%	10%	N	17.4%	10%	N
ASA classification	ASA II		15.8%	11.11%		9.3%	11.1%	N
	ASA III		71.7%	55.6%		72.1%	55.6%	
	ASA IV		12.5%	33.3%		18.6%	33.3%	

SS=statistical significance; Y=yes; N=no; ASA=American Society of Anesthesiologists; COPD=chronic obstructive pulmonary disease

**Fig. 1:**
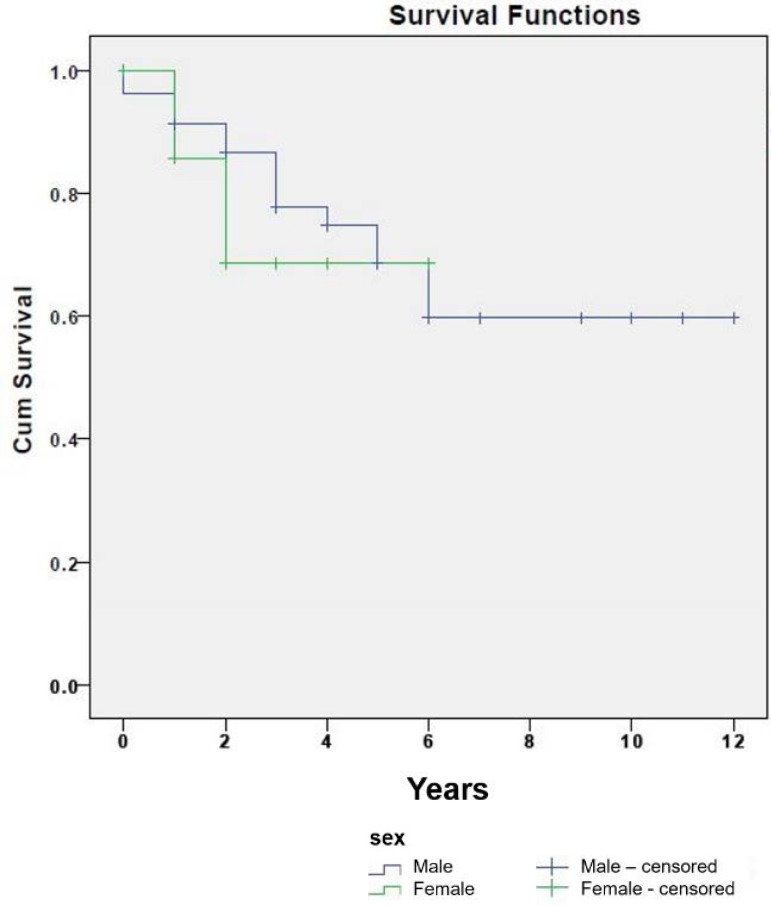
Survival curves by gender.

Detailed aneurysm characteristics are shown in Table 2. There was a statistical association between neck angulation >70º and women and a trend to statistical significance in the association of internal iliac aneurysm and aneurysm diameter >60 mm, in both groups. In group A, a trend to CIA diameter >20 mm in women that wasn't confirmed when adjusted by age (Group B) was observed.

**Table 2:** Comparison of aneurysm characteristics between women and men (Groups A and B).

		**Group A (general)**		**SS**	**Group B (adjusted by age)**		**SS**
		**Men (n=161)**	**Women (n=10)**		**Men (n=47)**	**Women (n=10)**	
Aneurysm Morphology	Aortic	67.2%	40%	N	68%	40%	N
	Unilateral Aortolliac	21.8%	40%		19.1%	40%	
	Bilateral Aortolliac	11.2%	20%		12.8%	20%	
IIA Aneurysm		8.9%	30%	0.07	8.5%	30%	0.09
Aneurysm Diameter		62.1mm	66.7mm	N	60.2mm	66.7mm	N
Aneurysm Diameter	<60 mm	48.3%	20%	0.08	48.9%	20%	0.092
	>60 mm	51.7%	80%		51.1%	80%	
Neck length	<10 mm	11.2%	0	N	15%	0	N
	>10 mm	88.8%	100%		85%	100%	
Neck diameter	<28 mm	85.7%	87.5%	N	97.5%	87.5%	N
	>28 mm	14.3%	12.5%		2.5%	12.5%	
Neck calcification	<50%	95.9%	87.5%	N	95.2%	87.5%	N
	>50%	4.1%	12.5%		4.8%	12.5%	
Neck thrombus	<50%	84.9%	87.5%	N	87.8%	87.5%	N
	>50%	15.1%	12.5%		12.2%	12.5%	
	None	18.3%	12.5%		9.5%	12.5%	
Neck angulation	<50°	48.6%	25%	N	50%	25%	N
	>50°	33.1%	62.5%		40.5%	62.5%	
	<70°	92.3%	62.5%	Y	92%	62.5%	Y
	>70°	7.7%	37.5%		7.1%	37.5%	
Neck shape	Conical	28.2%	25%	N	24.4%	25%	N
	Reversal Conical	4.9%	0		2.4%	0%	
	Cylindrical	63.4%	75%		73.2%	75%	
	Other	3.5%	0		0	0	
Iliac Tortuosity	Small/Medium	76.4%	40%	N	66.7%	40%	N
	Large	23.6%	60%		33.3%	60%	
Right CIA Diameter	<20 mm	75.2%	50%	0.051	77.5%	50%	N
	>20 mm	14.8%	50%		22.5%	50%	
Left CIA Diameter	<20 mm	75.2%	55.6%	0.056	82.5%	55.6%	N
	>20 mm	14.8%	44.4%		17.5%	44%	
Right EIA		8.7%	9.2%	N	8.8%	9.2%	N
Left EIA		8.5%	8.3%	N	8.5%	9.3%	N

IIA=internal iliac artery; CIA=common iliac artery; EIA=external iliac artery

The outcomes (anesthesic time, surgical time, need of blood transfusion, lenght of stay, type of endoleak, sac behaviour, intraoperative complications, surgical re-intervention, thirty-day complications, and thirty-day mortality) showed no statistical difference between gender in the two groups (Tables 3 to 6).

**Table 3:** Gender and anesthesic time, surgical time, need for blood transfusion, and length of stay (Groups A and B).

		**Anesthesic time (minutes)**		**ES**	**Surgical time (minutes)**		**ES**	**Need blood transfusion**	**ES**	**Length of stay (days)**		**SS**
		**Mean**	**SD**		**Mean**	**SD**				**Mean**	**SD**	
Group A	Men	174.6	64.7	N	103.2	49.5	N	21.3%	N	6.4	7.3	N
	Women	170.1	32.5		95.3	26.8		40.9%		5.5	2.4	
Group B	Men	179.4	48.5	N	105.8	34.6	N	25%	N	5.6	3.3	N
	Women	170.1	32.5		95.3	26.8		50%		5.5	2.4	

SS=statistical significance; Y=yes; N=no; SD=standard deviation

**Table 6:** Gender and intraoperative complications, reintervention, thirty-day complications and thirty-day mortality (Groups A and B).

		**Intraoperative**	**ES**	**Re-intervention**						**Thirty-day**	**SS**	**Thirty-day**	**SS**
		**complications**		**General**	**SS**	**Endoleak IA**	**SS**	**Endoleak IB**	**SS**	**complications**		**mortality**	
Group A	Men	23.6%	N	16.1%	N	4.3%	N	4.3%	N	22.7%	N	1.20%	N
	Women	20%		20%		0		10%		10%		0	
Group B	Men	25.5%	N	19.1%	N	14.3%	N	0	N	28.6%	N	2.10%	N
	Women	20%		20%		0		10%		10%		0	

SS=statistical significance; Y=yes; N=no

Kaplan Meier curves of postoperative survival by patient's gender show a better survival in men but without statistical significance. The median survival in men was 8.5 years, with a standard deviation of 0.5 (95% CI: 7.5-9.5), and 4.6 years in women, with a standard deviation of 0.83 (95% CI: 3-6.2). We can see both survival curves in Figure 1, and there was no statistical significance between them.

## DISCUSSION

The surgical indication for AAA is mainly based in two randomized trials^[7,8]^. These studies have shown the benefit of surgery to patients with aortic aneurysms over 5.5 cm, with criteria equal for both genders. However, in these studies there were 83% and 99.2% of males, and so it can be questioned if the treatment proposed can be extended for both genders. Likewise, the 3 cm diameter needed to diagnose an AAA must be questioned if it is suitable for both genders, considering that characteristics like height, body surface and weight are different between males and females.

The larger randomized prospective studies (EVAR-1^[9]^, DREAM^[10]^, OVER^[11]^, ACE^[12]^ and EVAR-2^[13]^) that compared open surgery to EVAR and are seen as references to AAA treatment have a male percentage of 91%, 93%, 99.3%, 100% and 76.8%, respectively. This huge difference makes us wonder if it is reasonable to extrapolate the results to women. Menezes et al. ^[14]^ also has demonstrated this discrepancy in a retrospective regional study, with a male percent of 91.21%.

Due to a lower prevalence of AAA in women, they are excluded from screening programs, but Wanhainen et al.^[15]^ suggest that the prevalence may be underestimated. They state that if the criteria to diagnose an AAA changes from 3 cm in diameter to 1.5 times the diameter of the normal infra-renal aorta, then the prevalence in the age group from 65 to 75 years would go from 16.9% to 12.9% in men and from 3.5% to 9.8% in women.

Mofidi et al.^[16]^ suggest that aneurysm growth is higher in women than in men (3.67 mm/year in women vs. 2.03 mm/year in men, *P*<0.001). The UK small aneurysm trial also revealed a risk of rupture four times higher in women, and a risk of rupture with lower diameters (5.0±0.8 cm in women vs. 6.0±1.4 cm in men, *P*=0.001).

In 2006, Dillavou et al.^[17]^ revealed that EVAR was done to 28% of the women with AAA and to 44.3% in men. It is supposed that a worse profile of anatomical conditions in women explained this difference.

As for 30-day mortality rate, it was reported to be higher in women undergoing elective surgery and in rupture. Norman & Powell's review stated that fatality after elective surgery was 35% to 50% higher in women^[2]^. Heller et al.^[3]^ reported a mortality rate after conventional surgery of 7.7% in women and 5.1% in men. Leon et al.^[4]^ confirmed this mortality difference after conventional surgery, with a mortality of 8.2% in women and 5.2% in men, and after EVAR this difference was even higher: 5.1% in women *vs.* 1.7% in men. In a meta-analysis, Grootenboer et al.^[5]^ gathered 61 studies for a total of 516.118 patients and showed that women with AAA had a higher mortality rate comparing to men in the elective treatment by conventional surgery (7.6% *vs.* 5.1%) and in EVAR (2.9% *vs.* 1.5%).

One should also account for the higher survival in women and their different physiology: until menopause women have hormonal protection; after, this protection is lost, and they have a higher incidence of cardiovascular disease comparing to men.

Starr & Halpern^[18]^ reviewed the current recommendations and recent literature, to help finding the differences between gender. Their review showed that women presented later, have a higher rupture rate, and underwent AAA treatment less than men, and with varied outcomes, with some studies showing similar short-term and long-term results between men and women. They concluded that it must be some genderspecific risks inherent to women, although pathophysiologic mechanisms responsible for the difference in AAA prevalence between gender have not been determined. They affirmed that it is reasonable to recommend routine screening for women older than sixty-five years who have ever smoked or who have a family history of AAA and the need of additional research to elucidate the reasons for differences between men and women.

Skibba et al.^[6]^, in a 14-year retrospective study with 2121 patients and 499 women (23.5%), found that women with AAA were older than men, have a higher frequency of cardiovascular risk factors, had greater risk of rupture at all size intervals and a fourfold increased frequency of rupture at <5.5 cm.

Preiss et al.^[19]^ searched the differences in late mortality between females undergoing elective EVAR for small and/or slow-growing AAAs compared with those who meet standard criteria at their institution. They analyzed thirty-six women (22% of treated patients) for a mean follow-up time of 37.2 months. Sixteen of the thirty-six women (44.4%) presented an AAA diameter smaller than 5.5 cm or a six-month growing rate shorter than 0.5 cm. This group had a higher late mortality (37.5% *vs.* 5%; *P*=0.03) and a trend toward increased reoperation rate. This fact might compromise the EVAR indication in women who do not meet the standard criteria.

Gloviczki et al.^[20]^ analyzed the outcome results of consecutive patients who underwent EVAR between 1997 and 2011 at a tertiary center, with a total of 934 patients (13% female). They concluded that women had an increased rate of complications and reinterventions, but not a significantly higher mortality.

Chung et al.^[21]^ analyzed 1380 consecutive patients who underwent elective EVAR from 1992 to 2012, of which 214 were women. They referred that women were older and had less cardiac disease, had shorter necks and more angulated, had less iliac aneurysms and they need more adjunctive arterial procedures, with more perioperative complications, longer length of stay and higher rate of endoleaks. Despite more complex aneurysm anatomy and more perioperative complications, aneurysmrelated deaths and overall survival was similar between genders.

Ayo et al.^[22]^, in a retrospective single institution review, showed women with greater neck angulation and higher percent of thrombus, but with advantage in incidence of type I endoleaks (3.5% in men *vs.* 0% in women, *P*=0.381) and overall reinterventions rate (11.3% in men *vs.* 0% in women, *P*<0.05).

In our experience, EVAR and open surgery were offered independently of the gender, women were older (*P*<0.05) and had fewer association with smoking, ischemic heart disease, and vascular neurological disease. Concerning the anatomical characteristics, women are more associated to neck angulation >70º. Besides these anatomical findings, there was no statistical difference in the outcomes of EVAR between the genders.

## CONCLUSION

Despite the absence of significant numbers conditioned by the small sample, and despite the inherent limitation of a retrospective non-randomized study, our study shows a clear difference in the clinical and anatomical characteristics of the aneurysms among men and women, but with the same results of EVAR. This should be explored in order to understand the influence of gender in the etiology of AAA.

**Table:** 

**Authors' roles & responsibilities**	
RM	Conception and design study; operations and/or trials performance; analysis and/or data interpretation; manuscript writing or critical review of its content; final manuscript approval
GT	Manuscript writing or critical review of its content; final manuscript approval
PO	Statistical analysis; final manuscript approval
LL	Operations and/or trials performance; final manuscript approval
CP	Operations and/or trials performance; final manuscript approval
RA	Final manuscript approval

## Figures and Tables

**Table 4:** Type of endoleak by gender (Groups A and B).

	**Group A**		**SS**	**Group B**		**SS**
	**Men**	**Women**		**Men**	**Women**	
No endoleak	59.4%	60%	N	68.90%	60%	N
Endoleak I or III	10.3%	0		8.90%	0	
Endoleak II	24.5%	30%		20%	30%	
Endoleak II + I/III	5.8%	10%		2.20%	10%	

SS=statistical significance; Y=yes; N=no

**Table 5:** Aneurysmal sac behaviour after EVAR (Groups A and B).

	**Group A**		**SS**	**Group B**		**SS**
	**Men**	**Women**		**Men**	**Women**	
Sac growth	10.9%	10%	N	14%	10%	N
Sac shrinkage	89.4%	90%		86%	90%	
0-5 mm	21.8%	20%		20.9%	20%	
5-10 mm	32.7%	20%		25.6%	20%	
10-15 mm	12.2%	20%		18.6%	20%	
15-20 mm	9.5%	10%		11.6%	20%	
20-25 mm	4.8%	20%		4.7%	20%	
25-30 mm	4.1%	0		2.3%	0	
> 30 mm	4.1%	0		2.3%	0	

SS=statistical significance; Y=yes; N=no
